# Quantitative Angiographic Hemodynamic Evaluation After Revascularization Surgery for Moyamoya Disease

**DOI:** 10.1007/s12975-020-00781-5

**Published:** 2020-02-13

**Authors:** Yu Chen, Li Ma, Shuo Yang, Jan-Karl Burkhardt, Junlin Lu, Xun Ye, Weijian Jiang, Zeguang Ren, Rong Wang, Xiaolin Chen, Yuanli Zhao

**Affiliations:** 1 Department of Neurosurgery, Beijing Tiantan Hospital, Capital Medical University, Beijing, 100070 China; 2 New Era Stroke Care and Research Institute, The Rocket Force General Hospital, Beijing, China; 3 Department of Neurosurgery, Baylor College of Medicine Medical Center, Houston, TX USA; 4 China National Clinical Research Center for Neurological Diseases, Beijing, China; 5 Stroke Center, Beijing Institute for Brain Disorders, Beijing, China; 6 Beijing Key Laboratory of Translational Medicine for Cerebrovascular Disease, Beijing, China; 7 Beijing Translational Engineering Enter for 3D Printer in Clinical Neuroscience, Beijing, China; 8 Department of Neurosurgery, University of South Florida, Tampa, USA; 9 Department of Neurosurgery, Peking University International Hospital, Peking University, Beijing, China

**Keywords:** Moyamoya disease, Combined bypass, Internal carotid artery, Hemodynamic change, Vessel remodeling

## Abstract

The corresponding hemodynamic changes of the internal carotid artery (ICA) after the revascularization surgery for moyamoya disease (MMD) remain unclear. The aim of this study was to analyze the hemodynamic changes of the ipsilateral ICA after the combined direct and indirect extracranial-intracranial (EC-IC) bypass. MMD patients undergoing combined EC-IC bypass were retrospectively reviewed. The mean transit time (MTT) of ICA was evaluated by color-coding angiography before revascularization and at follow-up. The MTT defined as the blood transit time between the end of cervical portion (C1) and the C7 segment of ICA. The clinical prognosis was assessed with Matsushima grading system, moyamoya vessel reduction system, and modified Rankin Scale (mRS). The correlation between hemodynamic parameter and prognosis was analyzed. Subgroup analysis was conducted between different presentations and different ages. Fifty-one patients were identified and the mean imaging follow-up interval was 5.5 months. The ICA-MTT was increased after the combined revascularization (*P* < 0.001) compared with contralateral ICA. Faster preoperative ICA-MTT was significantly associated with improved mRS in the ischemic group (*P* = 0.05). The increased ICA-MTT was significantly associated with favorable neoangiogenesis (*P* = 0.04), moyamoya vessel reduction (> 50%) (*P* = 0.023), and improved mRS score (*P* = 0.008). In subgroup analysis, the correlation in the ischemic subgroup and adult subgroup remained significant. In this cohort, the ICA-MTT increased after the combined EC-IC bypass, and there was a positive correlation between the increased blood transit time and favorable outcomes. Color-coding DSA proved to be useful as a quantitative and serial method to monitor postoperative courses after revascularization in MMD.

## Introduction

Moyamoya disease (MMD) is characterized by chronic, progressive stenosis, or occlusion of unknown causes that occurred in the distal internal carotid artery (ICA) and its branches, along with development of collateral vessels at the base of the brain [1]. The clinical onset of MMD includes cerebral ischemia and intracranial hemorrhage. The most effective treatment for MMD is extracranial-intracranial (EC-IC) revascularization surgery including direct bypass, indirect bypass, or combined bypass techniques [2, 3]. The direct bypass or the combined bypass can improve intracranial perfusion by instantly increasing cerebral blood flow while later de novo collateral vessels will form from the indirect/extracranial graft, and it is increasingly considered more effective than only indirect treatment strategies for MMD patients [4–6]. Previous studies have reported that neoangiogenesis in the surgical area, reduced moyamoya vessels and improved intracranial perfusion (examined by postoperative computed tomography perfusion [CTP]), could be found during follow-up [7]. However, reports on hemodynamic changes of ICA after revascularization are still scant [8]. Though most of the ICA is located outside the skull, its hemodynamic changes may indirectly reflect the intracranial external carotid-internal carotid conversion and clinical prognosis.

Quantitative color-coding digital subtraction angiography (DSA) is a fairly new technique flattening a conventional 2D DSA image enables vision of cerebrovascular flow in a single image and quantifies mean transit time (MTT) though time-density curves [9]. In our previous work, quantitative color-coding DSA has been used to evaluate the hemodynamics in brain arteriovenous malformations and vasospasm after aneurysmal subarachnoid hemorrhage [10, 11]. In this study, we applied color-coding DSA to quantitatively evaluate the hemodynamic changes of the ipsilateral ICA after the combined revascularization. The MTT between the end of cervical portion (C1) of ICA and the C7 segment after the anterior choroidal artery originated from the ICA was elected to reflect the hemodynamic characteristics of ICA. We further investigate the correlation between ICA-MTT changes and angiographic or clinical improvement, and subgroup analysis was carried out to clarify whether the correlation still exists in hemorrhagic and ischemic-onset MMD.

## Materials and Methods

### Study Design and Participants

The participants included in this study were from a single-center cohort of MMD patients treated between February 2016 and March 2017. A total of 176 consecutive patients diagnosed with MMD in Peking University International Hospital were reviewed including clinical records and radiological data. Written informed consent for collecting clinical information was obtained from each patient at admission. This study protocol was approved by the Institutional Review Board at Peking University International Hospital. The inclusion criteria were as follows: (1) Patients with diagnosis of MMD by DSA/MRA according to the guideline for MMD (criteria of the Research Committee on Spontaneous Occlusion of the Circle of Willis, 2012) [12]. (2) Patients’ preoperative and follow-up DSA examination was available. (3) Patients received only combined EC-IC bypass procedures; the reason for selecting this population was to exclude the influence of different surgical types on the establishment of collateral circulation. The surgical procedure type is an important but controversial factor and may have different influence on the imaging results. Exclusion criteria were as follows: (1) Patients with diagnosis of moyamoya syndrome with an identified cause. (2) Patients without preoperation or follow-up DSA. (3) Patients diagnosed as unilateral MMD.

Finally, a total of 51 patients were enrolled in this study. The onset manifestations were categorized into 2 main types: ischemic (cerebral infarction, transient ischemic attack [TIA], headache, and epilepsy) and hemorrhagic presentation. MMD patients without presenting ischemic or hemorrhagic symptoms were not treated in this cohort. All the clinical symptoms or the disease types manifested at the initial attack were determined by neurosurgeons with at least 5 years’ experience of clinical practice.

### Surgical Procedures

The indication for the revascularization was based on the guidelines set by the Japanese Ministry of Health and Welfare [12]. The symptomatic and hemodynamically affected hemisphere was the preferred side for revascularization surgery. If the patient’s symptoms were significantly improved after the first surgery and the patient did not have symptoms that could be ascribed to the contralateral hemisphere, operative treatment of the contralateral hemisphere was not carried out. Otherwise, surgery on the contralateral hemisphere was performed. In this study, all patients received unilateral surgery when performed the second DSA.

In this study, all of the enrolled patients were underwent combined bypass (direct + indirect bypass) [13]. The direct bypass involves the end-to-side anastomosis of one of the branches of the superficial temporal artery (STA) to the cortical branches of middle cerebral artery (MCA). The graft patency was routinely confirmed with intraoperative indocyanine green videoangiography or postoperative computed tomography angiography (CTA) (3–5 days after surgery). The indirect bypass in the combined bypass setting was an encephalo-duro-arterio-synangiosis (EDAS). For the EDAS, the other STA branch was sutured onto the arachnoid surface of the brain after being dissected free and in addition the dura mater is reversed and then attached to the surface of the brain.

### DSA Acquisition and Measurement

2D DSAs were available preoperatively and at follow-up for all participants in this study. All DSA procedures were performed with a single C-arm fluoroscopy system (Philips, Amsterdam, Netherlands). A standard angiographic method (transfemoral route) was applied in all patients, and image acquisition was performed via a 5F catheter with the tip positioned at the beginning of the ICA, about 2 cm distal to the common carotid artery (CCA) bifurcation. The 2D DSA series were acquired with a rate of 4 frames per second. For image acquisition, 5 ml of contrast material was automatic injected in all series by power injector at a flow rate of 3 ml/s for 1.67 s. The color-coded DSA was obtained with postprocessing software (syngo iFlow; Siemens, Berlin, Germany), with the minimum measurement accuracy of 0.26/0.27 s. For each set of manually drawn region of interest (ROI), the time versus intensity graph was produced automatically by the software with the following parameters [11]:

- ROI peak time: time that contrast intensity of selected ROI reached the peak value.
- ROI arrival time: time of arrival of contrast material.
- MTT: average contrast material transit time through the target.

**Fig. 1:**
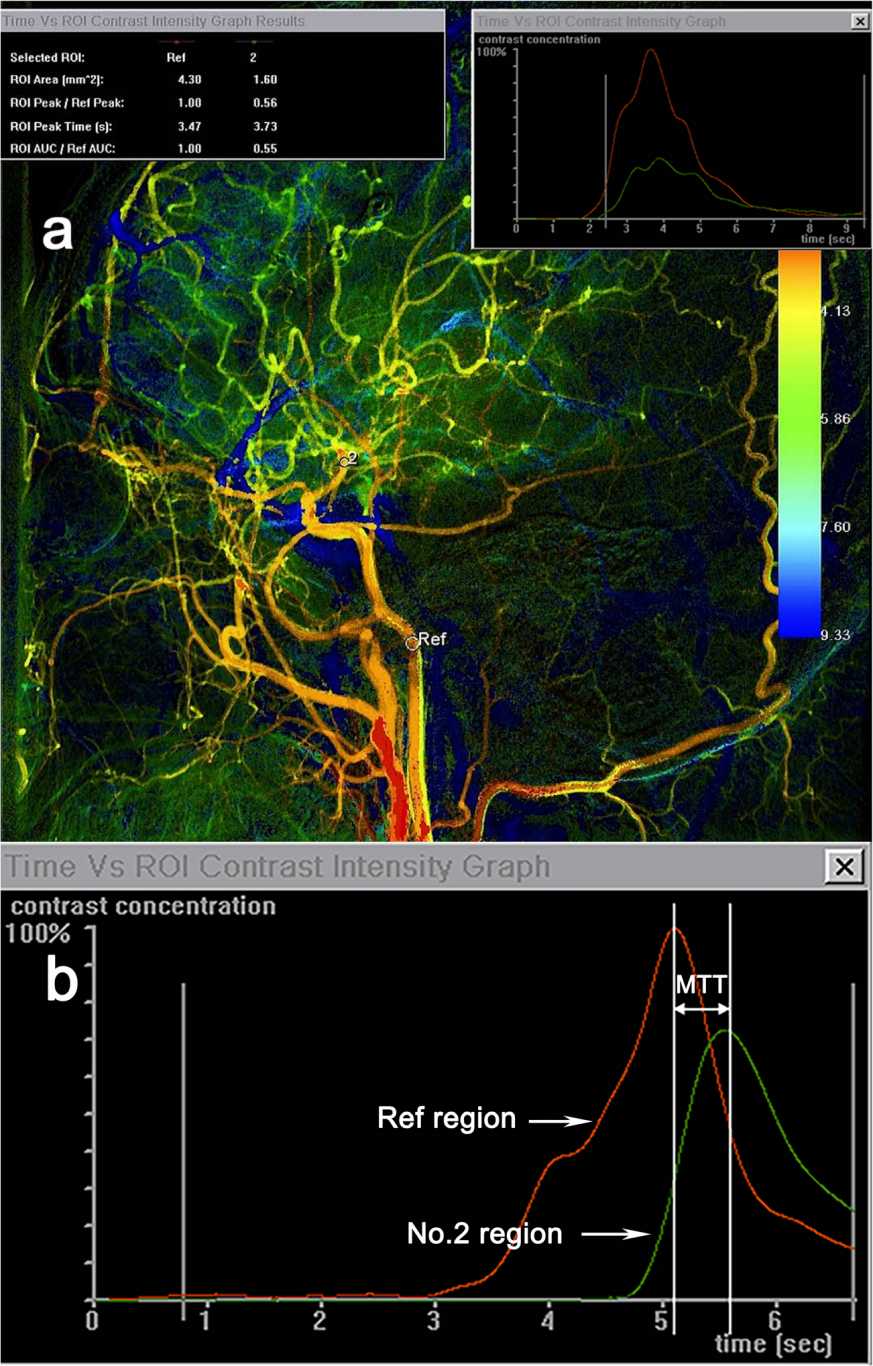
Color-coding digital subtraction angiography (DSA) for moyamoya disease (MMD) (sagittal position) and the measurement principle of hemodynamic parameter. Internal carotid artery-mean transit time (ICA-MTT) was defined as the difference of the region of interest (ROI) peak time intensity between the end of ICA C1 (Ref region, red line) and the C7 segment after the anterior choroidal artery originated from the ICA (No.2 region, green line) (**a**). Time-density curve showing MTT (**b**)

In this study, we focus on postoperative hemodynamic changes of ICA in MMD patients. The indirect index (MTT) was elected to reflect the transformation. One ROI was set at the end of cervical portion (C1) of ICA, and the other ROI was set at the C7 segment after the anterior choroidal artery originated from the ICA using the lateral view of color-coded DSA. The ROI’s diameter is the maximum diameter of the artery. ICA-MTT was defined as the difference between the two ROI peak times above (Fig. 1). The changed MTT (ΔICA-MTT) pre and post the combined bypass was calculated with the following formula:

$$ \Delta \mathrm{ICA}-\mathrm{MTT}=\mathrm{follow}-\mathrm{up}\ \mathrm{ICA}-\mathrm{MTT}\hbox{--} \mathrm{preoperative}\ \mathrm{ICA}-\mathrm{MTT}. $$

The contralateral hemodynamic parameters were also measured as the natural history control group. The measurement of iFlow data was conducted independently by at least 2 radiologists who are with at least 5 years of clinical experiences in radiology center of our institute. A series of training program was performed to ensure the accuracy of data measurement, such as the position they measured before and after the operation must be completely consistent.

### Angiographic and Clinical Outcomes After Surgery

DSA, CTP, and clinical follow-up were conducted 3–12 months later after the operation for postoperative patient when the patient came back to our hospital for review. The modified Rankin Scale (mRS) score was obtained on admission and in the follow-up. For pediatric patients, the neurological status was evaluated with child improved mRS classification [14]. The evaluation of mRS score was conducted by clinical neurosurgeons who have at least 5 years’ experience of clinical practice and a series of training program was performed to ensure the accuracy of mRS measurement. Researchers who performed follow-up assessments were blinded to the iFlow result and angiographic outcomes.

The postoperative angiographic outcomes of all cases were classified as three groups according to the criteria proposed by Matsushima [6]. This grading was the proportion of MCA area of distribution supplied by the surgical revascularization: grade A, more than two-thirds of the MCA distribution; grade B, between two-thirds and one-third of the MCA distribution; and grade C, less than one-third of the MCA distribution. We used moyamoya vessel reduction staging system [15] to classify the postoperative change of moyamoya vessels at the cranial base [15–17]. The method to evaluate numbers of MMD vessels was to measure the area with pathological basal vessels and calculate the relative percentage of the pathological MMD area compared with the area of the MCA feeding territory in the capillary phase of the anteroposterior view in the internal carotid angiogram. All cases are classified as three groups: grade 1, the MMD vessels significantly decreased (> 50%); grade 2, the MMD vessels not significantly decreased (25–50%); grade 3, no evidence of decrease (< 25%) or appear increase. The angiographic treatment outcomes were evaluated by 2 independent neuroradiologists (with at least 5 years’ experience of clinical practice) who were blinded to the clinical information.

### Statistical Analysis

The categorical variables are presented as counts (with percentages). Mean and standard deviation were used to describe the continuous variables with normal distribution, and median and inter-quartile were used to describe non-normal distribution continuous variables. Pearson chi-square test, Fisher exact test, or Mann-Whitney *U* test were used to compare categorical variables as appropriate. Wilcoxon rank sum test was applied to compare ICA-MTT in the surgical side preoperation and follow-up. Kruskal-Wallis *H* test were used to analyze the correlation among ΔICA-MTT with the angiographic scale and mRS. Group differences were analyzed by ANOVA variance analysis after case rank. *P* value < 0.05 was considered to be statistical significant. Statistical analysis was performed using SPSS (version 25.0, IBM, New York, USA).

## Results

### Baseline Characteristics

A total of 51 patients were included in the study. The patients’ mean age at the time of revascularization was 34.8 ± 12.7 years (ranging 6–57 years). In terms of age distribution, 5 pediatric patients and 46 adult patients were treated. Onset clinical symptoms were hemorrhagic in 9 cases and ischemic in 42 cases. The hemorrhagic patients were all adult patients. Analyzing the preoperative angiography, most of the patients were presented with Suzuki stage III (33.3%) and Suzuki stage IV (43.1%). The mean follow-up period of angiogram and neurological outcomes was 5.5 months (ranging 3–16.5 months) after the combined bypass (Table 1).

**Table 1:** Baseline characteristics and outcomes of ischemic and hemorrhagic MMD patients

Characteristic	All patients (*n* = 51)	Ischemic (*n* = 42)	Hemorrhagic (*n* = 9)	*P* value
Age (years)	34.8 ± 12.7	33.6 ± 13.5	40.1 ± 6.4	0.168
Detail presentation				
Infarction		23 (54.8)		
TIA		12 (28.6)		
Headache		4 (9.5)		
Epilepsy		3 (7.1)		
Admission mRS score	1.4 ± 0.7	1.4 ± 0.7	1.4 ± 0.5	0.952
Suzuki stage				
I–II	2 (3.9)	2 (4.8)	0 (0.0)	1.000
III	17 (33.3)	14 (33.3)	3 (33.3)	1.000
IV	22 (43.1)	16 (38.1)	6 (66.6)	0.230
V–VI	10 (19.6)	10 (23.8)	0 (0.0)	0.242
Perioperative complications				
Hyperperfusion syndrome	15 (29.4)	13 (31.0)	2 (22.2)	0.906
Infarction	4 (7.8)	4 (9.5)	0 (0.0)	0.778
Hemorrhage	2 (3.9)	1 (2.4)	1 (11.1)	0.781
Wound infection	1 (2.0)	1 (2.4)	0 (0.0)	1.000
TNE	11 (21.6)	11 (26.2)	0 (0.0)	0.198
Follow-up (months)	5.5 ± 2.5	5.4 ± 2.5	5.9 ± 3.0	0.798
Preoperative ICA-MTT (s)				
Median (IQR)	0.27 (0–0.54)	0.27 (0–0.54)	0.26 (0.13–0.67)	0.792
Follow-up ICA-MTT (s)				
Median (IQR)	0.54 (0.27–0.80)	0.54 (0.27–0.87)	0.54 (0.27–0.94)	0.764
ΔICA-MTT (s)				
Median (IQR)	0.26 (0–0.53)	0.16 (0–0.53)	0.27 (0.01–0.54)	0.432
Matsushima grading system				0.411
Grade A	13 (25.5)	11 (26.2)	2 (22.2)	1.000
Grade B	23 (45.1)	20 (47.6)	3 (33.3)	0.680
Grade C	15 (29.4)	11 (26.2)	4 (44.4)	0.492
MMD vessels diminishment grading system				0.592
Grade 1	25 (49.0)	22 (52.4)	3 (33.3)	0.503
Grade 2	12 (23.5)	8 (19.0)	4 (44.4)	0.231
Grade 3	14 (27.5)	12 (28.6)	2 (22.2)	1.000
Follow-up mRS score	0.8 ± 0.9	0.8 ± 0.9	0.8 ± 1.0	0.924
Improved mRS score	30 (58.8)	24 (57.1)	6 (66.7)	0.765

*ICA*, internal carotid artery; *IQR*, inter-quartile range; *MTT*, mean transit time; *MMD*, moyamoya disease; *mRS*, modified Rankin Scale; *TIA*, transient ischemic attack; *TNE*, transient neurological event

Values are numbers of cases (%) unless otherwise indicated. Mean values are presented with standard deviation

### Hemodynamic Changes After Combined Revascularization Surgery

**Fig. 2:**
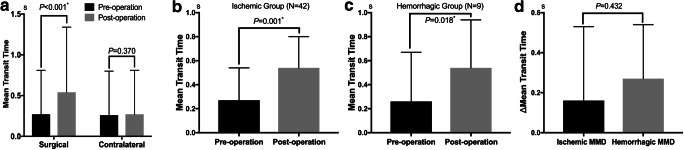
Comparison of internal carotid artery-mean transit time (ICA-MTT) before the operation and follow-up: The ICA-MTT was significantly increased after surgery (**a**); In subgroup analysis, the prolongation was still existed in both the hemorrhagic and the ischemic group (**b, c**); The ΔICA-MTT in the hemorrhagic group also has no significant differences with the ischemic group (**d**). *P value indicates statistical significance

The postoperative ICA-MTT of the surgical side was significantly increased at follow-up (*P* < 0.001), with a baseline ICA-MTT of 0.27 s and ICA-MTT at follow-up of 0.54 s. These changes were not observed in the internal control group of the contralateral ICA (*P* = 0.370) (Fig. 2). Additionally, there was a significant difference in ΔICA-MTT between the two groups (*P* < 0.001) (Table 2).

**Table 2:** Baseline characteristics and hemodynamic parameters of surgical and contralateral side

Characteristics	Surgical side (*n* = 51)	Contralateral side (*n* = 51)	*P* value
Suzuki stage			
Mean ± SD	3.8 ± 0.8	3.5 ± 1.0	0.114
I–II	2 (3.9)	7 (13.7)	0.163
III	17 (33.3)	17 (33.3)	1.000
IV	22 (43.1)	19 (37.3)	0.545
V–VI	10 (19.6)	8 (15.7)	0.603
Preoperative ICA-MTT (s)			
Median (IQR)	0.27 (0–0.54)	0.26 (0–0.54)	0.851
Follow-up ICA-MTT (s)			
Median (IQR)	0.54 (0.27–0.80)	0.27 (0.26–0.54)	0.035*
ΔICA-MTT (s)			
Median (IQR)	0.26 (0–0.53)	0 (0–0.26)	0.002*

*ICA*, internal carotid artery; *IQR*, inter-quartile range; *MTT*, mean transit time; *SD*, standard deviation

Values are numbers of cases (%) unless otherwise indicated. Mean values are presented with SDs

*Statistical significance (*P* < 0.05)

In subgroup analysis, the postoperative ICA-MTT was significantly increased both in the ischemic group (*P* = 0.001) and the hemorrhagic group (*P* = 0.018). The ΔICA-MTT in ischemic group was similar to that of hemorrhagic group (Fig. 2). In addition, the prolongation was more significant in the adult patients (*P* < 0.001). The ΔICA-MTT was similar between adults and children (Table 3).

**Table 3:** Hemodynamic parameters and outcomes of adults and children

Characteristics	All patients (*n* = 51)	Adults (*n* = 46)	Children (*n* = 5)	*P* Value
Preoperative ICA-MTT (s)				
Median (IQR)	0.27 (0–0.54)	0.27 (0–0.54)	0.27 (0–0.8)	0.939
Follow-up ICA-MTT (s)				
Median (IQR)	0.54 (0.27–0.80)	0.53 (0.27–0.735)	1.07 (0.27–1.07)	0.361
ΔICA-MTT (s)				
Median (IQR)	0.26 (0–0.53)	0.06 (0–0.405)	0.52 (0.27–0.63)	0.145
Matsushima grading system				0.079
Grade A	13 (25.5)	10 (21.7)	3 (60.0)	0.185
Grade B	23 (45.1)	21 (45.7)	2 (40.0)	1.000
Grade C	15 (29.4)	15 (32.6)	0 (0.0)	0.316
MMD vessels diminishment grading system				0.169
Grade 1	25 (49.0)	22 (47.8)	3 (60.0)	0.963
Grade 2	12 (23.5)	10 (21.7)	2 (40.0)	0.719
Grade 3	14 (27.5)	14 (30.4)	0 (0.0)	0.357
Follow-up mRS score	0.8 ± 0.9	0.8 ± 0.9	0.8 ± 1.0	0.597
Improved mRS score	30 (58.8)	26 (56.5)	4 (80.0)	0.593

*ICA*, internal carotid artery; *IQR*, inter-quartile range; *MTT*, mean transit time; *MMD*, moyamoya disease; *mRS*, modified Rankin Scale

Values are numbers of cases (%) unless otherwise indicated. Mean values are presented with standard deviation

### Postoperative Angiographic and Clinical Outcomes

After a mean follow-up of 5.5 months, 13, 23, and 15 patients were classified as grades A, B, and C when followed-up according to Matsushima neoangiogenesis grading system, respectively. In terms of moyamoya vessel reduction, 25, 12, and 14 patients were classified as grades 1, 2, and 3 when followed-up, respectively. The postoperative neoangiogenesis and the reduction of moyamoya vessels were similar in patients with ischemic-onset MMD and hemorrhagic-onset MMD (neoangiogenesis, *P* = 0.411; moyamoya vessel diminishment, *P* = 0.592).

The mRS scores of 30 patients were improved and no patients had worsened mRS scores compared with the baseline. There was no significant difference between the neurological improvement of patients with ischemic and hemorrhagic MMD (*P* = 0.765) (Table 1).

### The Correlation Between Preoperative ICA-MTT/ΔICA-MTT and Outcomes

The preoperative ICA-MTT has no significant correlation with postoperative neoangiogenesis and moyamoya vessel reduction. There was a trend that faster preoperative ICA-MTT related with improved mRS score, but not statistically significant (*P* = 0.065). In subgroup analysis, the correlation between faster preoperative ICA-MTT and improved mRS score in the ischemic group was at a statistically critical value (*P* = 0.05), but the correlation was not significant in the hemorrhagic group. In addition, the correlation between faster preoperative ICA-MTT and improved mRS score was statistical significant in the adult subgroup (*P* = 0.047).

**Fig. 3:**
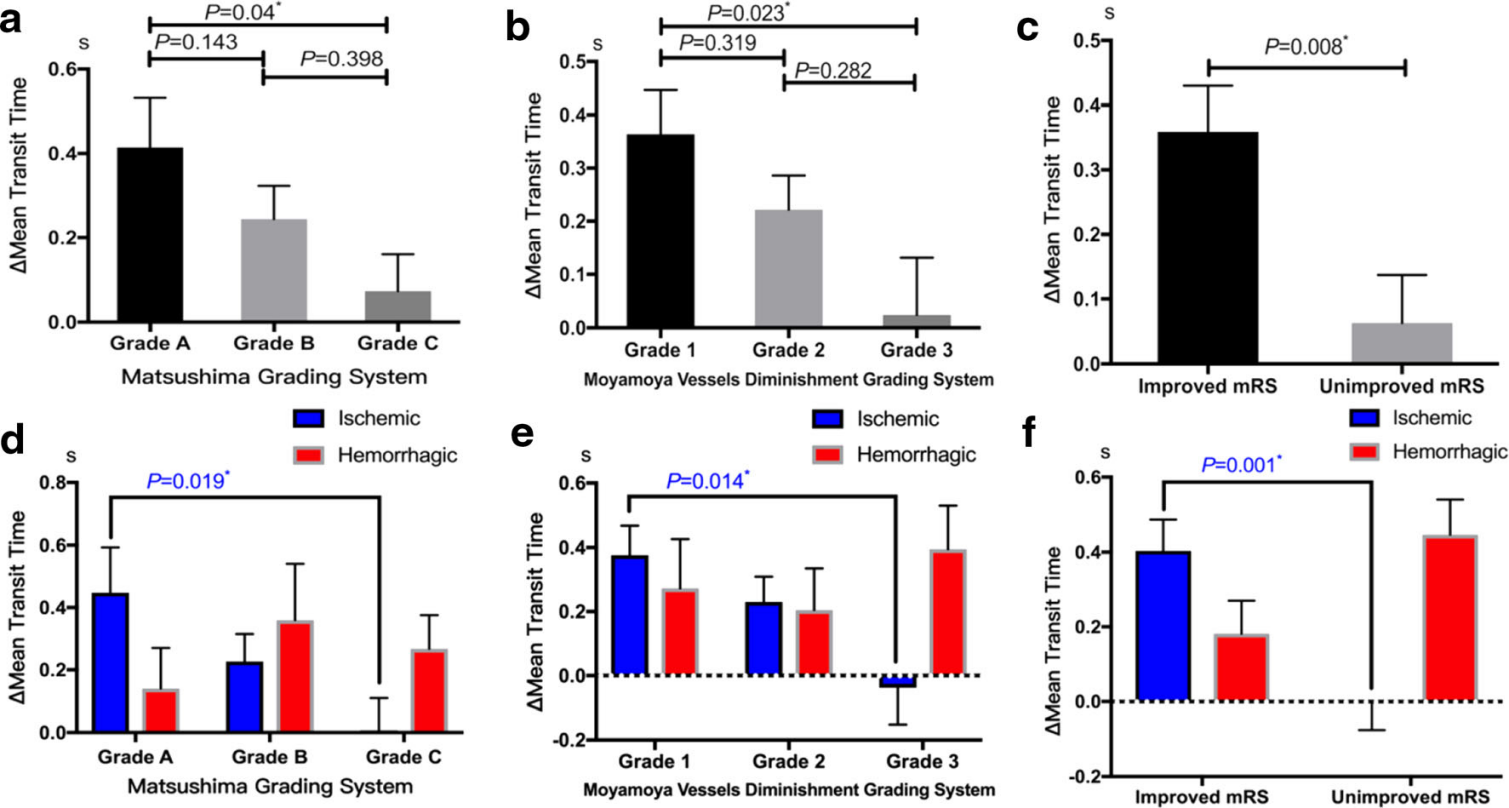
The correlation between ΔICA-MTT and angiographic and clinical outcomes in the overall study population and subgroup: Longer ΔICA-MTT was associated with better neoangiogenesis (Matsushima grade A), more moyamoya vessel diminishment (grade 1), and improved mRS scores in the overall study population (**a, b, c**). This correlation remained obvious in the ischemic group (**d, e, f**). *P value indicates statistical significance (*P* < 0.05)

The ICA-MTT prolonged more obvious in patients with the most favorable neoangiogenesis (Matsushima grade A), compared with that of patients with less neoangiogenesis (Matsushima grade C) (*P* = 0.04). In terms of moyamoya vessel diminishment, the prolonged ICA-MTT of patients with the most obvious moyamoya vessel reduction (grade 1) was more significant than patients with less moyamoya vessel reduction (grade 3) (*P* = 0.023). In subgroup analysis, the correlation between ΔICA-MTT and neoangiogenesis or the reduction of moyamoya vessels remained significant in the ischemic group (neoangiogenesis: *P* = 0.019, moyamoya vessel diminishment, *P* = 0.014). The prolonged ICA-MTT was more significant in patients with an improved mRS score after combined bypass surgery (*P* = 0.008), and this correlation remained significant in the ischemic group (*P* = 0.001) (Fig. 3). Besides, in the age-based subgroup analysis, the prolonged ICA-MTT was significantly related with improved mRS score in the adult subgroup (*P* = 0.034) than in the children subgroup (*P* = 0.157).

## Case Illustration

**Fig. 4:**
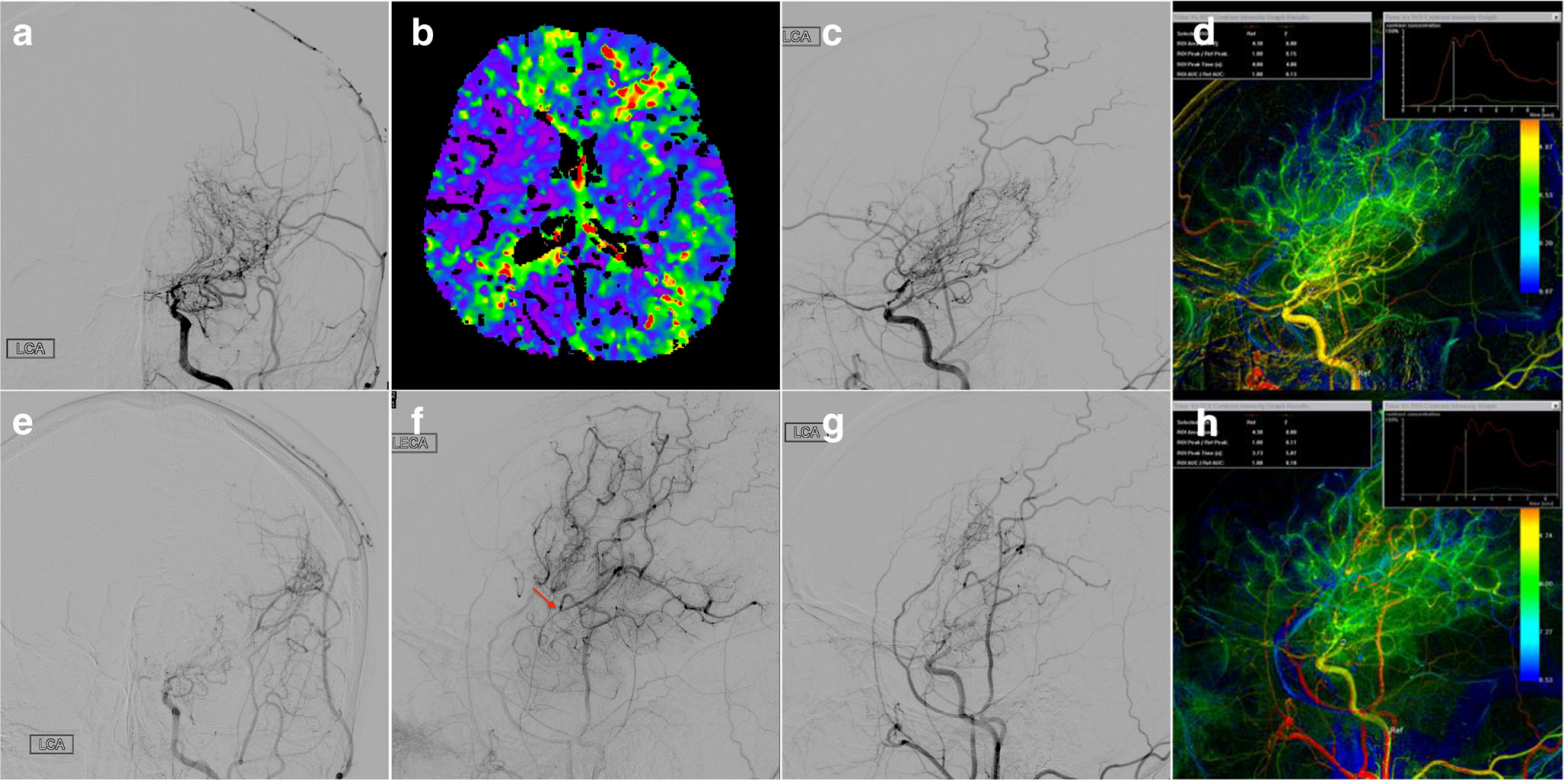
Preoperative digital subtraction angiography (DSA) of the left hemisphere showed Suzuki stage III moyamoya disease (MMD) and large amount of moyamoya vessels (**a**). Cerebral blood flow (CBF) of computed tomography perfusion (CTP) showed poor perfusion in the left middle cerebral artery (MCA) territories, especially in the left frontal lobe (**b**). The preoperative blood flow in the internal carotid artery (ICA) was fast, and the ICA-MTT was 0 s (**c**, **d**). Six months later, the moyamoya vessels of the left hemisphere were significantly reduced (**a** vs **e**). Good angiogenesis was observed and evaluated as Matsushima grade A (anastomotic site: red arrow) (**f**). The postoperative blood flow in the ICA was significantly prolonged to 1.34 s (**g**, **h**)

A 23-year-old male presented with paroxysmal numbness of right upper limb and speech disability for 1 week before admission. Preoperative DSA revealed Suzuki stage III in the left hemisphere. Preoperative MRI (7 days before the surgery) showed no acute infarcts. CTP showed decreased perfusion in the left MCA territories, especially in the left frontal lobe. Three days after admission, the patient underwent left-sided combined EC-IC revascularization bypass procedure. No neurological deficits occurred postoperatively and 6 months later, the patient returned for clinical follow-up and received cerebral angiography. In the quantitative color-coding DSA, the preoperative blood flow in the ICA on the surgical side was fast with an ICA-MTT less than minimum measurement accuracy (< 0.26/0.27 s). During follow-up, the ICA-MTT was significantly prolonged to 1.34 s. Meanwhile, obvious new conducted collateral vessels in the MCA territory (Matsushima grade A) and conspicuous reduced moyamoya vessels (grade 1) were detected (Fig. 4). Neurologically the patient was intact (mRS of 0) at 6-month follow-up and his previously described right upper extremity numbness and speech disorder symptoms disappeared.

## Discussion

Revascularization bypass techniques have been routinely performed on MMD patients for flow augmentation and improved perfusion [18]. With the formation of collateral vessels, a series of intracranial angioarchitectural changes after bypass surgery have been observed, including diminished moyamoya vessels, reversal of dilated anterior choroidal and posterior communicating arteries (AChA-PCoA), and occlusion of associated miliary aneurysms [15, 19–21]. These angioarchitectural characteristics are originated from ICA and the postoperative changes suggested that the hemodynamics of ICA may experience corresponding changes after the revascularization. In this study, we investigated the ICA hemodynamic changes using quantitative iFlow color-coding angiography before revascularization and at postoperative follow-up. We found that the ICA-MTT in the surgical side significantly increased during follow-up after EC-IC revascularization bypass. Additionally, these changes were associated with the angiogenesis of collateral vessels, the diminishment of moyamoya vessels, and the improvement of neurological outcomes.

The prolonged ICA-MTT indicated that the ICA had experienced vascular remodeling after the combined bypass. One previous study reported that the volumetric flow and pressure drop of the ICA will decrease after bypass surgery for MMD, and they also suggested that there was a good correlation between the angiographic grading and the pressure drop index [8]. However, this technique requires re-examination of 3D-MRA which is not routinely performed after the operation and this study did not set up a control group to exclude the mixed effects of MMD natural history. Previous studies identified that the vascular remodeling of ICA and vessels of Willis circle could also be the natural history of MMD [22–25]. In the histological investigation of autopsy specimens of MMD, the stenosis or occlusion with fibrocellular intimal thickening and the attenuation of the media at the circle of Willis have been observed [23, 26–29]. Not only that, the outer diameter of the circle of Willis was indicated experience diminishment at the disease progresses, especially in the MCA [23–25]. In this study, we measured the contralateral ICA as the natural history control group to distinguish the confounding effect of natural course of MMD on the hemodynamic changes of ICA. The contralateral ICA-MTT was found slightly prolonged after the combined bypass during the follow-up, but there was no statistical difference. Preoperative ICA-MTT was almost the same in both sides, but the ICA-MTT of the surgical side was significantly prolonged than contralateral side during the follow-up. We recognized that the ICA of the surgical side will experience drastic hemodynamic changes in the short term after the combined bypass, which are more likely to be caused by revascularization than by natural history changes. The decreased demand of blood supply from ICA and the increased perfusion from the new collaterals after bypass surgery might explain the ICA remodeling and corresponding hemodynamic changes in MMD [30, 31].

Bypass surgery has been demonstrated to prevent ischemic and hemorrhagic stroke in MMD patients by increasing cerebral blood flow to the ischemic brain through the newly collateral circulation constructed from the EC artery [17, 32]. And several studies have been suggested that the degree of collateral circulation angiogenesis has a significant correlation with postoperative prognosis [6, 33]. In addition, the improvements in the anterior choroidal artery dilation and decreases in moyamoya vessels have been hypothesized to be relevant angiographic changes following bypass [15, 17]. In this study, we found that most patients appeared corresponding improvement of angiopathy and clinical prognosis, and the improvement has a good correlation with the prolonged ΔICA-MTT. Therefore, the ΔICA-MTT may be used as a quantitative index during serial observation. In previous studies, ischemic-onset, abundant ICA moyamoya vessels, Suzuki stages 3–4, and preoperative neurological status were reported to be associated with better neoangiogenesis and more favorable long-term outcome after revascularization surgery for MMD [6, 34–36]. However, these factors were not quantitative and mainly based on the preoperative features, which would be inappropriate for serial evaluation. The present study suggested a consecutive quantitative parameter (ICA-MTT) to evaluate the therapeutic effects in an objective manner. In the subgroup analysis, the present data indicated that the correlations between ΔICA-MTT and angiopathy improvement or neurofunctional outcomes were more stable in the ischemic group, comparted with the hemorrhagic group. However, it should be noted that the small sample size of hemorrhagic group (*n* = 9) might not provide a conclusive result on this issue.

At present, accumulating evidence indicates that surgical revascularization may be the most effective treatment paradigm for ischemic MMD [37–39]. Our previous studies compared the long-term postoperative neurofunctional outcomes of different revascularization strategies and suggested that direct bypass was more effective in preventing recurrent ischemic strokes than indirect bypass for ischemic MMD, although neurological function outcomes were similar [40–42]. However, the treatment options for hemorrhagic MMD remain controversial [17, 43, 44]. The mechanisms underlying hemorrhagic MMD have not been deeply understood and involve interactions between hemodynamic stress and thin arterial walls. It have been reported that the dilation of AChA-PCoA, the fragile moyamoya vessels, and the presentation of complicated periventricular miliary aneurysms are associated with the rupture event of hemorrhagic MMD [45–49]. Theoretically, the effect of revascularization surgery to increase the intracranial perfusion and reduce the burden of moyamoya vessels may help to decrease the rate of recurrent hemorrhagic stroke. Several studies indicated that the combined bypass could reduce the risk of re-bleeding by improving the dilated ICA branches (AChA-PCoA) and promoting the spontaneous occlusion of the flow-related aneurysms [15]. However, many studies hold the opposite view, and they detected no differences in long-term mortality and angiographic improvement during follow-up between patients with hemorrhagic MMD who underwent bypass and those who received conservative therapy [20, 50]. In this study, we found that the ICA-MTT was obviously prolonged both in the ischemic group and hemorrhagic group after the combined bypass and there was no significant difference in ΔICA-MTT between the two groups. The increased ICA-MTT and corresponding angiopathy improvement might indicate a reduced stress on the ICA branches and moyamoya vessels, and thereby provide hemodynamic evidence for the decreased risk of re-bleeding.

## Limitation

This observational study was limited by its small sample size, limited surgical strategies, and measurement errors. First, our hemorrhagic sample size was relatively small, which reduces the power to detect meaningful differences between subgroups. Second, our findings are based on the patients who received combined revascularization surgery. Therefore, further studies would be required to determine whether the present findings remained significant in the overall MMD patients with different bypass procedures (direct and indirect bypass). Additionally, because of the C7 segment after the takeoff of anterior choroidal artery is enclosed in the skull base, artifacts of the skull on DSA may lead to inaccurate peak time measurements in the ROI. Therefore, future investigations with larger sample size and various surgical strategies would be required.

## Conclusion

In this cohort, the blood flow transit time of ICA increased after combined direct/indirect EC-IC bypasses for MMD. The prolonged blood flow transit time of the ICA is correlated with favorable neoangiogenesis, more moyamoya vessel diminishment, and improvement of neurological outcomes, especially in the ischemic patient subgroup. Color-coding DSA may provide quantitative and serial hemodynamic parameter (ICA-MTT) for evaluating postoperative outcomes of MMD after revascularization surgery. Larger cohorts and different surgical strategies are needed to be analyzed in the future.
